# Novel Toll-like receptor-4 antagonist (+)-naloxone protects mice from inflammation-induced preterm birth

**DOI:** 10.1038/srep36112

**Published:** 2016-11-07

**Authors:** Peck Yin Chin, Camilla L. Dorian, Mark R. Hutchinson, David M. Olson, Kenner C. Rice, Lachlan M. Moldenhauer, Sarah A. Robertson

**Affiliations:** 1 Robinson Research Institute and Adelaide Medical School, University of Adelaide, Adelaide, SA 5005, Australia; 2 Adelaide Medical School, University of Adelaide, Adelaide, SA 5005, Australia; 3 Australian Research Council Centre of Excellence for Nanoscale BioPhotonics, Adelaide, SA, 5005, Australia; 4 Departments of Obstetrics & Gynecology, Pediatrics and Physiology, University of Alberta, Edmonton, Alberta T6G2S2, Canada; 5 Chemical Biology Research Branch, National Institute on Drug Abuse and National Institute on Alcohol Abuse and Alcoholism, National Institutes of Health, Rockville, MD 20892, USA

## Abstract

Toll-like receptor 4 (TLR4) activation by bacterial infection, or by sterile inflammatory insult is a primary trigger of spontaneous preterm birth. Here we utilize mouse models to investigate the efficacy of a novel small molecule TLR4 antagonist, (+)-naloxone, the non-opioid isomer of the opioid receptor antagonist (−)-naloxone, in infection-associated preterm birth. Treatment with (+)-naloxone prevented preterm delivery and alleviated fetal demise *in utero* elicited by i.p. LPS administration in late gestation. A similar effect with protection from preterm birth and perinatal death, and partial correction of reduced birth weight and postnatal mortality, was conferred by (+)-naloxone administration after intrauterine administration of heat-killed *E. coli*. Local induction by *E. coli* of inflammatory cytokine genes *Il1b*, *Il6, Tnf* and *Il10* in fetal membranes was suppressed by (+)-naloxone, and cytokine expression in the placenta, and uterine myometrium and decidua, was also attenuated. These data demonstrate that inhibition of TLR4 signaling with the novel TLR4 antagonist (+)-naloxone can suppress the inflammatory cascade of preterm parturition, to prevent preterm birth and perinatal death. Further studies are warranted to investigate the utility of small molecule inhibition of TLR-driven inflammation as a component of strategies for fetal protection and delaying preterm birth in the clinical setting.

Preterm birth, defined as birth at less than 37 weeks of gestation, accounts for 12% of all births worldwide,varying from 5–13% according to geographic region, race and socioeconomic status^1^,^2^. The cost to infant health is enormous, particularly in undeveloped nations where the majority of spontaneous premature birth occurs^3^. Preterm birth is now the major cause of death in children under 5 years, accounting for 1.1 million deaths annually^4^, while surviving infants are at risk of a raft of developmental impairments with lifelong consequences^5^,^6^. Even late preterm infants born at 34–36 weeks gestation have substantial mortality and neonatal morbidity, and have elevated risk of long-term cardiovascular and metabolic conditions^5^,^7^. New interventions are urgently needed to tackle the underlying causes of preterm birth and stem the impact on fetal injury and ensuing child health^8^.

Preterm delivery (PTD) results when the labour and birth processes of normal parturition are prematurely activated. On-time, term labour involves events characteristic of a progressively accelerating inflammatory cascade^9^. Fetal signals trigger a surge in pro-inflammatory cytokines including *Il1b*, *Il6* and *Tnf* within the fetal membranes, uterus and cervix and an accompanying influx of leukocytes into the gestational tissues^10^,^11^ amplifies the inflammatory sequalae to promote cervical ripening and rupture of the fetal membranes^12^,^13^. Cytokines interact with hormone changes to mediate induction of uterine activation genes (UAGs) and prostaglandin signaling, culminating in uterine contractions and expulsion of the fetus^14^,^15^.

Precocious inflammation leading to preterm labor can be triggered by infection or by sterile pro-inflammatory insults, originating either within gestational tissues or systemically, from tissues distal to the placenta and fetus^1^,^16^. Detection of infection is mediated by sensing molecules known as Toll-like receptors (TLRs) that bind conserved molecular structures carried by microorganisms, known as pathogen-associated molecular patterns (PAMPs). TLRs also sense silent inflammation resulting from non-infectious insult, through ligation of endogenous agents released by injured tissue (damage-associated molecular patterns, DAMPs). The archetypal receptor in this detection system is TLR4, which is activated by the bacterial cell wall component lipopolysaccharide (LPS). TLR4 is extensively expressed throughout the female reproductive tract including the fetal membranes^17^,^18^ and placental trophoblasts^19^,^20^, as well as leukocytes, epithelial and mesenchymal cells in the cervix^20^ and uterus^21^. TLR-driven pathways are involved in normal on-time parturition, since mice with null mutation in *Tlr4* or *Tlr2* exhibit a delay in the timing of term labour^22^,^23^.

Infection-induced preterm labor can be modelled in mice by administration of LPS systemically or locally into the gestational tissues^24^,^25^,^26^, or delivery of killed *E. coli* into the uterine cavity or fetal membranes to mimic ascending infection and chorioamnionitis^27^,^28^. TLR4 is a key sensor for infection-induced preterm birth, since protection is afforded in mice by spontaneous mutation of *Tlr4* in C3H/HeJ mice^27^,^29^,^30^, null mutation of *Tlr4* in C57Bl/6 mice^22^,^30^, and genetic deficiency in the MyD88 and TRIF adaptor proteins required for TLR4 signaling^28^. TLR4 inhibitors including a lipid A mimetic and anti-TLR4 neutralising antibodies have previously shown promise in attenuating preterm birth in mouse infection models^24^,^30^, but neither of these are suitable for human clinical application.

These observations underpin our hypothesis that TLR4 inhibition with a novel TLR4 antagonist (+)-naloxone might comprise a useful strategy for targeted suppression of inflammation underlying preterm birth. (+)-Naloxone is a newly identified TLR4 antagonist that is the non-opioid isomer of the opioid receptor antagonist (−)-naloxone^31^, a well-described nonselective antagonist of the μ-opioid receptor commonly prescribed for opioid addiction, including in pregnant women and neonates^32^. (+)-Naloxone binds MD2 to prevent LPS engagement with TLR4^33^ leading to diminished immune NFκB activation and IL1B, IL6 and TNF synthesis^34^,^35^,^36^. In contrast to anti-TLR4 neutralizing antibodies^24^, (+)-naloxone is a small molecule which can penetrate across the placenta^37^, with a pharmacokinetic profile affording it short systemic exposure or longer term delivery if required. (−)-Naloxone has anti-inflammatory activity and provides protection against sepsis in animal models^38^,^39^. (+)-Naloxone has similar properties but unlike (−)-naloxone, does not have opioid receptor-binding activity, and is a specific antagonist of TLR4 signaling^31^.

In view of the attractive pharmacological properties of (+)-naloxone as a TLR4 antagonist and our previous observation that (+)-naloxone can delay the timing of natural on-time parturition^22^, we sought to investigate the possible utility of (+)-naloxone as a novel anti-inflammatory tocolytic agent. Here, we use two common mouse models of infection-induced preterm birth to show (+)-naloxone protects against preterm delivery and alleviates *in utero* and postnatal loss induced by TLR-driven inflammation.

## Results

### (+)-Naloxone prevents LPS-induced preterm delivery (PTD)

**Figure 1:**
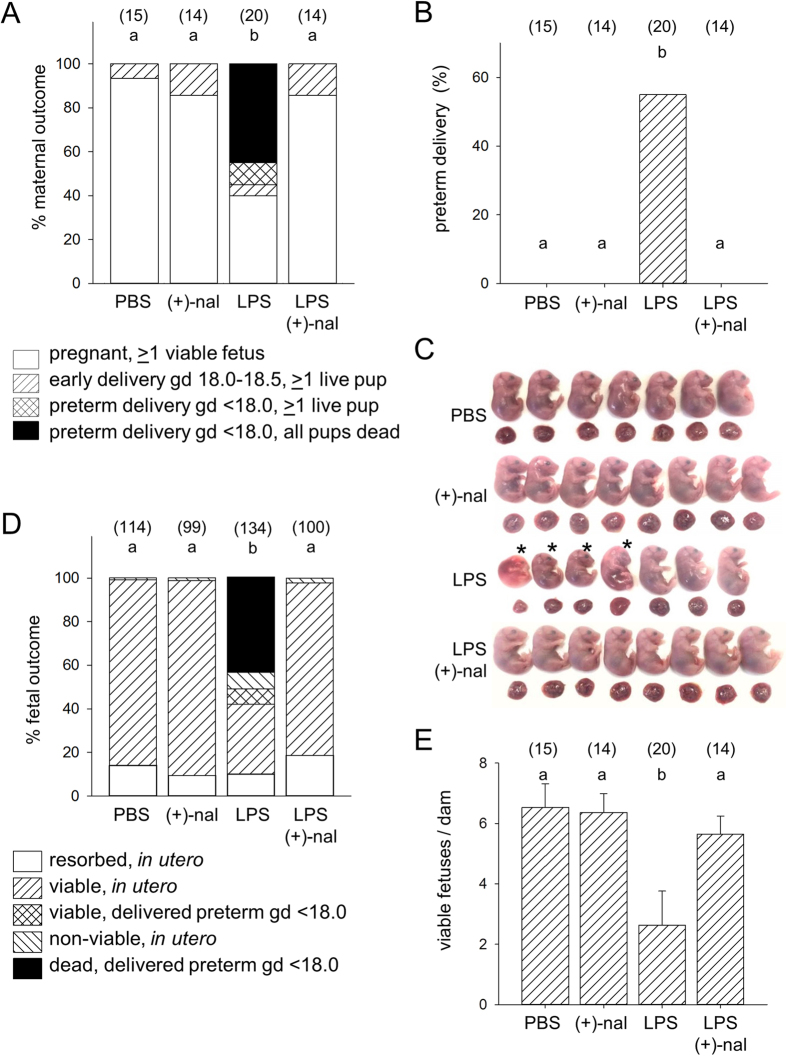
(+)-Naloxone prevents LPS-induced PTD and fetal loss. Pregnant B6 mice were administered LPS or PBS i.p. on gd 16.5, followed by (+)-naloxone or PBS i.p. on gd 16.5, 17.0, 17.5 and 18.0, then observed for PTD (delivered gd < 18.0) and autopsied at gd 18.5. **(A)** Dam outcomes at gd 18.5, classified as (i) pregnant (>1 live fetus); (ii) delivered early (gd 18.0–18.5); delivered preterm (gd < 18.0; >1 live pup), or (iv) delivered preterm, all pups dead. **(B)** The percentage of pregnant dams exhibiting PTD. **(C)** Fetuses and placentae dissected from a representative dam in each treatment group. Pups marked ‘*’ are judged non-viable. **(D)** Fetal outcomes at gd 18.5, classified as (i) resorbed; (ii) viable, in utero; (iii) viable, delivered preterm; (iv) non-viable, in utero, or (v) dead, delivered preterm. All pups born alive before gd 18.0 were dead within 36 h. **(E)** The number of viable pups per dam. Data in **(A,B,D)** are percentage analyzed by χ^2^ test. Data in **(E)** are mean ± SEM analysed by two-way ANOVA. Number of pregnant dams per group is shown in parentheses. ^a,b,c^Different letters indicate differences between groups, *p *< 0.05.

To investigate the effect of (+)-naloxone in a TLR4-dependent model of infection-associated preterm birth, pregnant B6 dams were administered a low dose of LPS (0.5 μg i.p.), selected after titration of LPS in pilot studies to elicit ~50% PTD, as previously described^40^,^41^. LPS was administered at gestation day (gd) 16.5 (~3 days before expected on-time birth at gd 19.5), followed immediately by the first of 4 doses of (+)-naloxone or vehicle control at 12 h intervals until gd 18.0. Mice given LPS without (+)-naloxone exhibited adverse outcomes, with a majority delivering dead pups (9/20) or live pups (2/20) prematurely within 36 h (and generally within 24 h) of LPS administration (Fig. 1A). Those few pups delivered alive before gd 18.0 failed to survive beyond 36 h. In all, PTD (defined as delivery of live or dead pups before gd 18.0) occurred in 55% (11/20) LPS-treated, but 0% (0/15) PBS-treated mice (*p* < 0.001) (Fig. 1B). In the 45% (9/20) LPS-treated dams remaining undelivered at gd 18.5, autopsy revealed a higher incidence of severe fetal growth restriction and fetal death (18.9%, 10/53) compared to PBS-treated controls (1/115, 0.8%, *p* = 0.023) (Fig. 1C,D). Taking into account pups delivered dead or identified as non-viable *in utero*, there was a 58% reduction in the mean number of viable fetuses per pregnant dam, compared with PBS-treated mice (Fig. 1E).

Treatment with (+)-naloxone protected pregnancy from LPS challenge, completely abrogating PTD (0/14 mice delivered before gd 18.0) (Fig. 1A,B). The low survival rate of fetuses *in utero* after LPS challenge was also reversed by (+)-naloxone, such that the mean number of viable fetuses per dam was not less than the control PBS-treated group (Fig. 1C–E). Moreover, administration of (+)-naloxone alone in the absence of LPS did not induce PTD (0/14 mice) or affect fetal survival rates (Fig. 1A–E).

**Figure 2:**
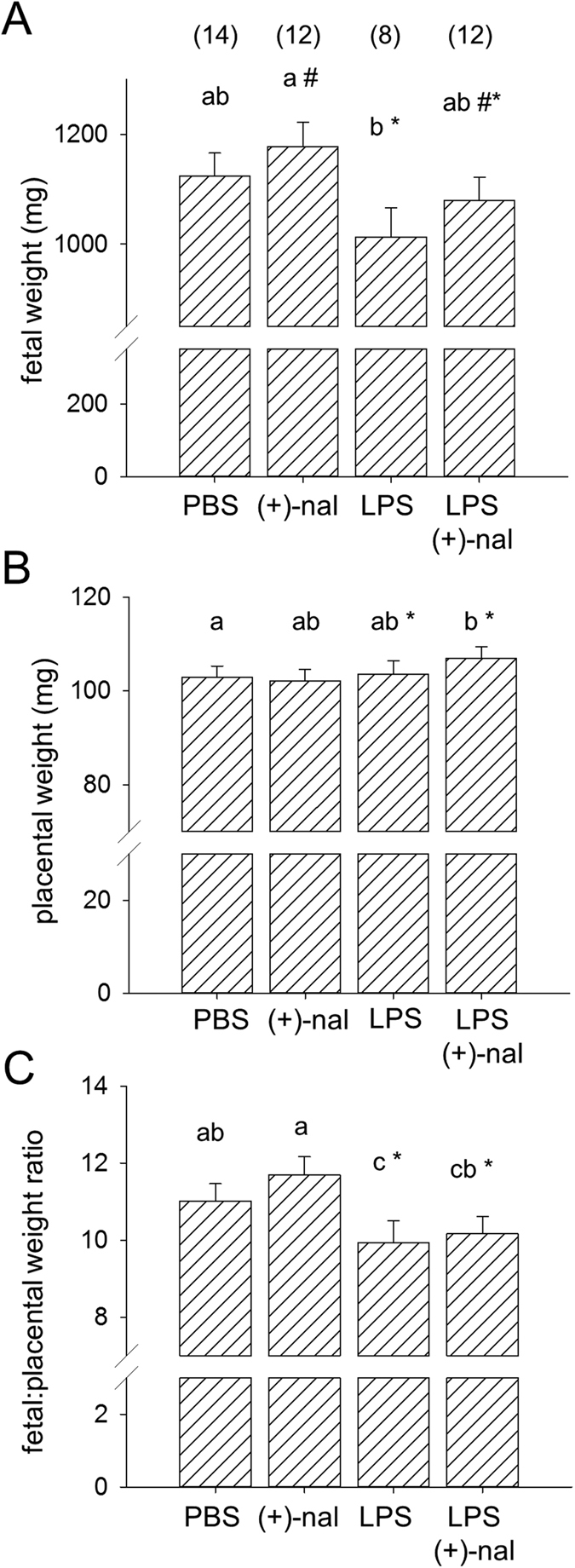
Effects of LPS and (+)-naloxone on fetal and placental weight. Pregnant B6 mice were administered LPS or PBS i.p. on gd 16.5, followed by (+)-naloxone or PBS i.p. on gd 16.5, 17.0, 17.5 and 18.0, then fetal weight, placental weight and fetal:placental weight ratio were measured at gd 18.5. **(A)** Fetal weight; **(B)** placental weight, and **(C)** fetal:placental weight ratio for surviving fetuses. Data are estimated marginal mean ± SEM, analyzed by Mixed Model Linear Repeated Measures ANOVA and post-hoc LSD t-test, with mother as the subject, adjusted for litter size. ^a,b,c^Different letters indicate differences between groups, *p *< 0.05. *Significant effect of LPS, independent of (+)-naloxone, *p* < 0.05. ^#^Significant effect of (+)-naloxone, independent of LPS, *p* < 0.05. Number of pregnant dams per group is shown in parentheses.

When fetuses surviving *in utero* at gd 18.5 were analysed for effects of treatments on fetal and placental weight, mixed model ANOVA showed that LPS reduced fetal weight (*p* = 0.033) (Fig. 2A) and elevated placental weight (*p* = 0.019) (Fig. 2B), causing a substantial reduction in fetal:placental weight ratio (*p* = 0.001) (Fig. 2C), a parameter indicative of placental functional efficiency. Conversely (+)-naloxone exerted a positive impact on fetal weight (*p* = 0.045), and did not affect placental weight or fetal:placental weight ratio. Post-hoc analysis to compare individual treatment groups demonstrated a 15.2% reduction in fetal:placental weight ratio after LPS treatment compared to PBS control (*p* = 0.036) (Fig. 2C), but (+)-naloxone treatment was not able to correct this.

These data indicate that (+)-naloxone can effectively inhibit TLR4 signaling to prevent PTD and rescue fetal loss elicited by LPS administration in late gestation. However, fetuses that survive LPS challenge *in utero* exhibited altered placental efficiency indicated by reduced fetal:placental weight ratio, regardless of (+)-naloxone treatment.

### (+)-Naloxone prevents heat-killed E. coli-induced preterm delivery

**Figure 3:**
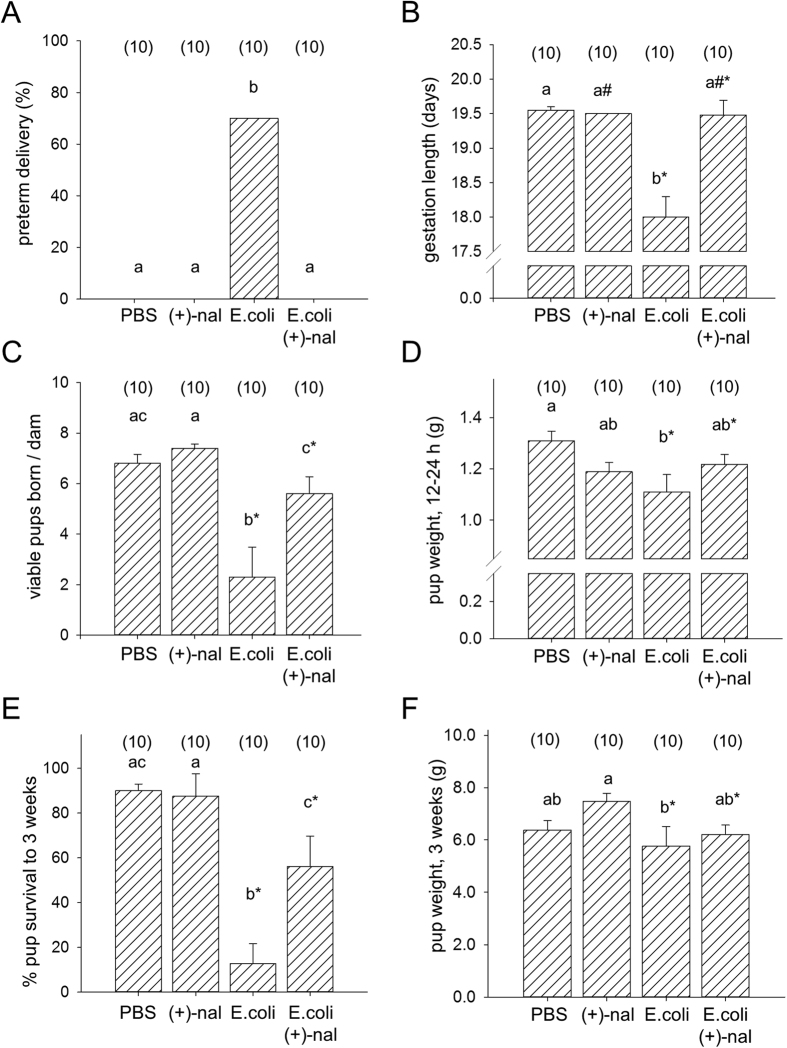
(+)-Naloxone prevents *E. coli*-induced PTD and fetal loss. Pregnant B6 mice were administered intrauterine heat-killed *E. coli* or PBS on gd 16.5, followed by (+)-naloxone or PBS i.p. on gd 16.5, 17.0, 17.5 and 18.0, then observed for PTD and allowed to progress to birth. **(A)** The percentage of pregnant dams exhibiting PTD, analyzed by χ^2^ test. **(B)** The gestation length, **(C)** number of viable pups born per dam, **(D)** pup weight at 24 h, **(E)** pup survival to 3 weeks and **(F)** weight of surviving pups at 3 weeks. **(B,C,E)** Data are mean ± SEM analyzed by two-way ANOVA, and **(D,F)** data are estimated marginal mean ± SEM, analysed by Mixed Model Linear Repeated Measures ANOVA and post-hoc LSD t-test, with mother as the subject. ^a,b,c^Different letters indicate differences between groups, *p* < 0.05. *Significant effect of *E. coli*, independently of (+)-naloxone, *p* < 0.001. Number of pregnant dams per group is shown in parentheses.

Given the ability of (+)-naloxone to prevent LPS-induced PTD, we next investigated whether (+)-naloxone can also prevent PTD induced by intact bacteria, which elicit PTD by ligation of multiple TLRs including TLR4 and TLR2^42^,^43^. In this experiment, we used a proven model of chorioamnionitis-induced PTD wherein heat-killed *E. coli* are surgically instilled into the uterine cavity between gestation sacs^27^,^28^. The dose of heat-killed *E. coli* was selected to elicit ~50% PTD after titration of *E. coli* in pilot studies. After *E. coli* administration on gd 16.5, mice were monitored for time of delivery and the survival of pups. Instillation of killed *E. coli* into the uterus on gd 16.5 caused PTD in 70% (7/10) of treated mice (Fig. 3A), with an associated 36 h average reduction in gestation length (Fig. 3B) and 66% reduction in viable litter size at birth (Fig. 3C). The timing of birth and live birth rate were substantially rescued by (+)-naloxone treatment, with none of 10 mice delivering before gd 18.0, a comparable gestation length, and no change in mean number of viable pups born compared to PBS control (Fig. 3A–C). Again, (+)-naloxone alone had no impact on timing of birth or litter size (Fig. 3A–C).

When surviving delivered pups were analysed for effects of treatments at 12–24 h after birth, mixed model ANOVA showed *E. coli* administration had a strong negative impact on pup birth weight (*p* < 0.001). (+)-Naloxone did not affect birth weight independently, but a significant interaction with *E. coli* was present (*p* < 0.001). Post-hoc analysis revealed that co-administration of (+)-naloxone alleviated the reduction in birth weight caused by *E. coli* treatment, such that the pups born after exposure *in utero* to both (+)-naloxone and *E. coli* were not different in weight to pups of PBS-treated control dams (Fig. 3D).

The majority of pups born small were lost in the post-natal and pre-weaning period, with only 13% of pups born alive from dams administered *E. coli* surviving to 3 weeks of age, compared to 90% and 88% respectively of pups from dams given PBS or (+)-naloxone controls. Co-administration of (+)-naloxone rescued the majority of pups with 56% surviving to 3 weeks of age (Fig. 3E), at which time their growth was comparable to those of the control groups (Fig. 3F).

This experiment demonstrates the utility of the TLR4 antagonist (+)-naloxone in preventing prematurity and perinatal death, and alleviating fetal and postnatal growth impairment, in a physiologically relevant model of preterm birth and *in utero* inflammation.

### (+)-Naloxone abrogates fetal and uterine cytokine expression induced by heat-killed E. coli

**Figure 4:**
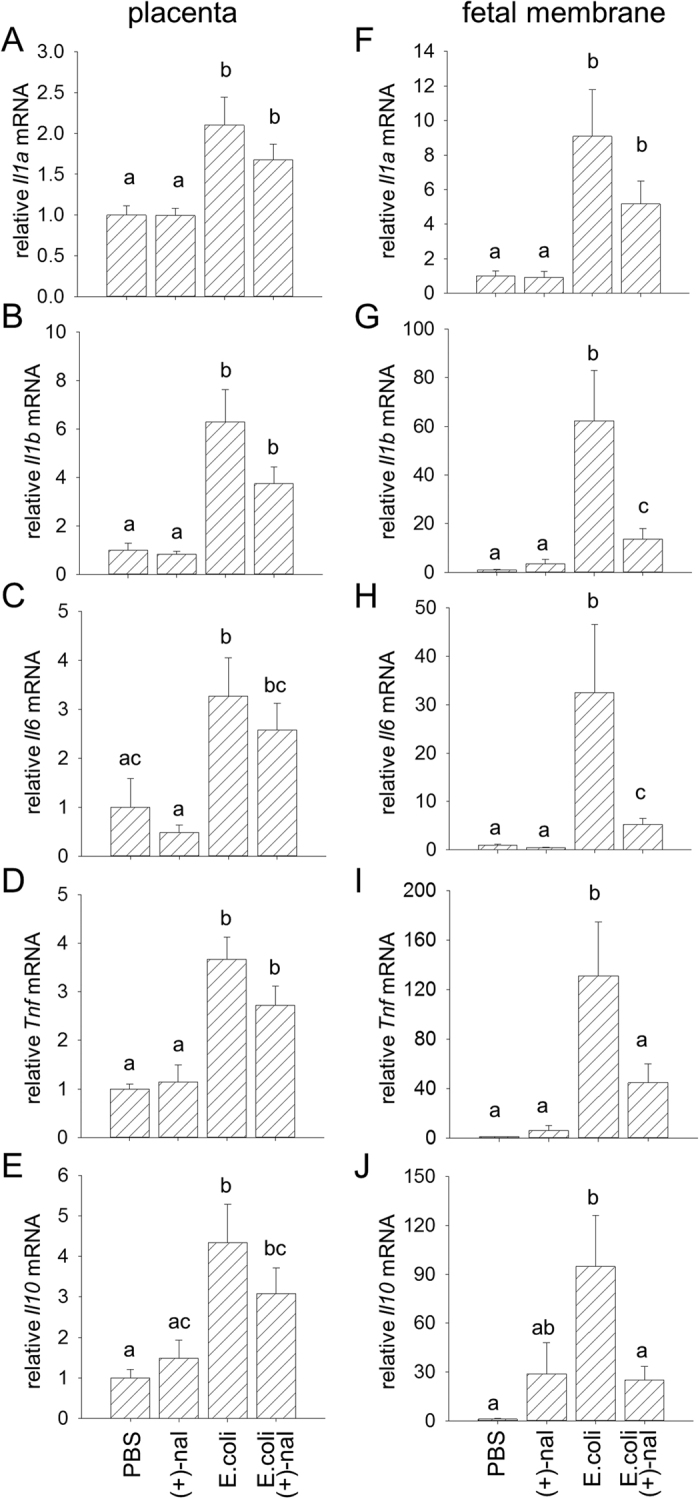
(+)-Naloxone suppresses *E. coli*–induced pro-inflammatory cytokine expression in placenta and fetal membranes. Pregnant B6 mice were administered intrauterine heat-killed *E. coli* or PBS on gd 16.5, followed by (+)-naloxone or PBS i.p., and 4 hours later, placenta and fetal membranes were recovered from the two adjacent implantation sites. Relative expression of *Il1a* **(A,F)***, Il1b* **(B,G)***, Il6* **(C,H)**, *Tnf* **(D,I)** and *Il10* **(E,J)** mRNAs were determined in placenta **(A–E)** and fetal membranes **(F–J)** by qPCR and normalised to *Actb*. Data are mean ± SEM relative gene expression of n = 12 tissues from n = 6 dams/group and were analysed by one-way ANOVA and post-hoc Sidak t-test. ^a,b,c^Different letters indicate differences between groups, *p* < 0.05.

*E. coli*-induced preterm birth is elicited when ligation of TLRs activates an inflammatory cascade, initiated when activation of NFκB triggers expression of pro-inflammatory cytokines *Il1b*, *Il6* and *Tnf* in the fetal membranes and other gestational tissues^22^,^28^,^44^. In turn, cytokines induce uterine activation genes to cause prostaglandin synthesis and signaling, cervical ripening and uterine contractions^15^,^45^. To examine the mechanisms underlying the protective action of (+)-naloxone, we evaluated whether pro-inflammatory cytokines induced by intrauterine instillation of *E. coli* are influenced by (+)-naloxone administration. The placenta and fetal membranes, as well as maternal decidua and myometrium were collected from the two fetal units located immediately adjacent to the injection site, 4 h after *E. coli* administration with or without (+)-naloxone treatment, as before. In the fetal membranes and placenta, the sites of earliest response to TLR ligation to trigger the labor cascade^22^, *E. coli* induced a 9.1-fold and 2.1-fold increase in *Il1a* expression, a 62-fold and 6.3-fold increase in *Il1b*, a 33-fold and 3.3-fold increase in *Il6*, a 131-fold and 3.7-fold increase in *Tnf*, and 95-fold and 4.3-fold increase in *Il10*, compared to tissues from control mice given PBS (Fig. 4A–J). Administration of (+)-naloxone substantially reduced *E. coli*-driven cytokine expression particularly in fetal membranes, meeting statistical significance for *Il1b, Il6, Tnf* and *Il10*, which were reduced by 78%, 84%, 66% and 73% respectively (Fig. 4G–J).

**Figure 5:**
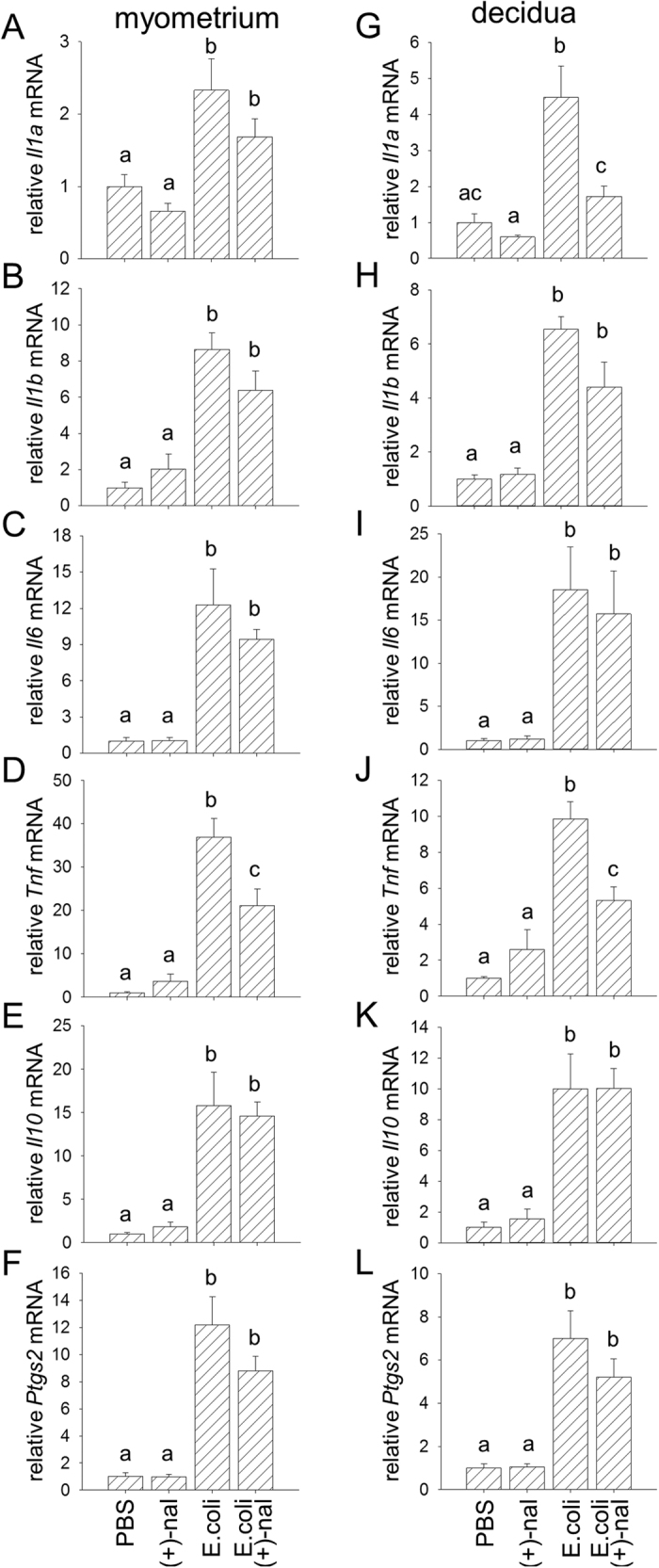
(+)-Naloxone suppresses *E. coli*–induced pro-inflammatory cytokine expression in uterine decidua and myometrium. Pregnant B6 mice were administered intrauterine heat-killed *E. coli* or PBS on gd 16.5, followed by (+)-naloxone or PBS i.p., and 4 hours later, uterine decidua and myometrium were recovered from the two adjacent implantation sites. Relative expression of *Il1a* **(A,G)***, Il1b* **(B,H)***, Il6* **(C,I)**, *Tnf* **(D,J),** *Il10* **(E,K)** and *Ptgs2* **(F,L)** mRNAs were determined in placenta **(A–F)** and fetal membranes **(G–L)** by qPCR and normalised to *Actb*. Data are mean  ± SEM relative gene expression of n = 12 tissues from ne = 6 dams/group and were analysed by one-way ANOVA and post-hoc Sidak t-test. ^a,b,c^Different letters indicate differences between groups, *p* < 0.05.

In the maternal tissues of the uterine decidua and myometrium, which are more spatially distant from the injection site, *E. coli* elicited increases in some of the same pro-inflammatory cytokines, although to a lesser extent than in fetal tissues (Fig. 5A–L). (+)-Naloxone given with *E. coli* challenge reduced decidual expression of *Il1a* by 62% (Fig. 5G), and reduced *Tnf* in both the myometrium and decidua by 43% and 45% respectively (Fig. 5D–J). Most uterine activation genes (*Gja1, Oxtr, Ptger4, Ptgfr* and *Ptgs1*; data not shown) were not elevated by *E. coli* treatment, likely because of the short 4 h time window between treatment and tissue analysis. However, *Ptgs2* encoding prostaglandin-endoperoxide synthase 2 (cyclooxygenase-2, Cox2) was increased in response to *E. coli* challenge in both the myometrium (12.1-fold, Fig. 5F) and decidua (7.0-fold, Fig. 5L) compared to control. A trend towards decreased *Ptgs2* after (+)-naloxone treatment in *E. coli* challenged mice did not reach statistical significance in either tissue (Fig. 5F,L). (+)-Naloxone administered without *E. coli* had no impact on gene expression in any of the fetal or maternal tissues (Figs 4 and 5).

These data show that the surge in pro-inflammatory cytokines released from fetal tissues contacting killed *E. coli* to trigger PTD, is suppressed by (+)-naloxone. This is consistent with an action of (+)-naloxone in preventing TLR4 ligation to trigger initiation of the inflammatory cascade that ultimately leads to preterm birth.

## Discussion

Normal on-time delivery is an inflammatory process that involves release of endogenous TLR ligands from the maturing fetus to ligate TLRs in fetal membranes and placental tissue^22^,^23^,^46^. Preterm delivery results when infection and/or sterile inflammatory agents cause pathogen-derived or endogenous TLR ligands to prematurely activate the TLR-induced inflammatory cascade^8^,^26^. Here we demonstrate that administration of a novel small molecule TLR4 antagonist (+)-naloxone is an effective treatment to suppress PTD and associated perinatal death induced in mice by systemic LPS or intrauterine administration of heat-killed *E. coli*. Gene expression analysis reveals that (+)-naloxone treatment inhibits *E. coli*-induced progression of the labor cascade by inhibiting induction in fetal membranes of pro-inflammatory cytokines *Il1a*, *Il1b*, *Il6* and *Tnf*, which are identified as responsible for the amplification of inflammation and eventual maturation of the myometrium to overcome quiescence, induce cervical ripening, activate uterine contraction and expel the fetus^41^,^47^. All dams that received four doses of (+)-naloxone at 12 h intervals, effectively encompassing the window between inflammatory challenge and earliest gestational age compatible with post-delivery survival in mice, delivered on time with the expected number of pups. The majority of pups survived the perinatal period and developed normally to 3 weeks of age. This demonstrates that (+)-naloxone protection of fetuses from TLR4-driven inflammation in late gestation is consistent with normal birth, postnatal survival and development of offspring at least to weaning age.

Exposure to TLR4-induced inflammation impacted fetal growth in late gestation, in association with evidence of reduced placental transport function, as described in previous studies^40^,^48^. Although no protective effect on mean fetal weight in viable fetuses was discernible at gd 18.5, fetuses at this time had been exposed to a pro-inflammatory environment for just 48 h, and effects on growth were less evident than in the *E. coli* model where birth weights were examined. Reduced birth weight was evident in pups exposed *in utero* to *E. coli*, and this was at least partly reversed by (+)-naloxone treatment.

TLR4 is present in many compartments of the gestational tissues and reproductive tract and is well-positioned as a key sensing system and rapid first-line response to ascending or systemic bacterial infection. Fetal membrane TLR4 expression appears of paramount importance as the membranes comprise the site of earliest response to endogenous TLR ligation to trigger term labor^22^. The highest fold-change expression in inflammatory cytokines induced by *E. coli* was seen in this tissue as opposed to placental or uterine tissues, and additionally cytokine suppression by (+)-naloxone was most profound in this compartment.

Mouse models of preterm birth often report heterogeneous effects amongst dams including preterm delivery of dead and live pups, and/or fetal injury or death *in utero* without delivery^48^,^49^. In the current study most dams delivered dead pups within 36 h of LPS or *E. coli* administration. With this outcome it is not obvious whether fetal death occurs *in utero* or in the perinatal phase, and thus whether death is the cause or consequence of early delivery. However, the observation of fetal death *in utero* in several dams, 48 h after LPS administration at gd 18.5, is consistent with the view that fetal injury and death can occur *in situ* after inflammatory challenge, independently of early delivery. Likewise, birth of live pups in a small proportion of preterm deliveries suggests fetal death is not essential for premature activation of the maternal parturition response. Consistent with previous reports^48^, we found that live pups born before gd 18.0 died soon after birth, presumably due to a combination of developmental immaturity and inflammatory injury inflicted *in utero*. These observations are consistent with interacting but distinct causal pathways underpinning the effects of inflammatory triggers on fetal and maternal tissue compartments.

Suppression of TLR4-induced pro-inflammatory cytokine expression in gestational tissues is inferred to be the key mechanism underlying the failure of bacterial agent-induced inflammation to progress to premature labor in mice given (+)-naloxone, in turn protecting mice from subsequent late gestation or perinatal death of fetuses. Our data using both LPS and heat-killed *E. coli* to induce preterm delivery are consistent with previous studies employing these models^27^,^28^, where elevated IL1B, IL6 and TNF signaling are implicated in progressing both the uterine maturation and injury to fetal tissues that ultimately results in preterm birth and fetal death respectively.

Administration of (+)-naloxone to mice given *E. coli* substantially suppressed cytokine expression, particularly in fetal membranes and variably in placenta, the tissues most proximal to *E. coli* deposition between fetal sacs. This demonstrates that inhibition of TLR4 signaling in these tissues can prevent the progression of the inflammatory cascade that culminates in preterm birth and fetal death. It is notable that (+)-naloxone is efficacious despite the fact that *E. coli* activates inflammation by activation of TLRs in addition to TLR4, including TLR2^42^,^43^. Different TLRs can trigger different cellular responses in gestational tissues, for example in placental trophoblasts where TLR4 ligation induces cytokine expression whereas TLR2 predominantly leads to apoptosis^19^. This presumably affects placental function and fetal growth, thus explaining why despite robust protection from preterm birth and perinatal death, (+)-naloxone was only partially effective in mitigating fetal growth impairment and protecting growth restricted pups from death in the postnatal phase following *E. coli* treatment *in utero*. Although (+)-naloxone is a small molecule that is able to penetrate the placental barrier^37^, it is possible that (+)-naloxone accesses the maternal and fetal compartments to differing extents, perhaps reaching higher and more effective concentrations in maternal tissues than in fetal tissues. Further experiments are required to investigate this, as well as to evaluate how (+)-naloxone affects cervical ripening, another important aspect of the parturition cascade.

In addition to pro-inflammatory genes, we examined effects of *E. coli* and (+)-naloxone on expression of UAGs. Induction of UAGs is secondary to hormone changes and pro-inflammatory cytokine upregulation^15^ so given the short time frame of the gene expression experiment, it is unsurprising that most UAGs were not elevated 4 h following *E. coli* administration. Expression of *Ptgs2* which controls PGE2 synthesis was elevated 4 h after *E. coli* administration, and a trend to suppression in its expression was evident with (+)-naloxone treatment. (+)-Naloxone has previously been shown to reduce LPS-induced TLR4-mediated *Ptgs2* and PGE2 synthesis in mouse macrophages^36^. PGE2 is a key factor in the prostaglandin signaling pathway required for cervical ripening and parturition^50^. Sustained delivery of (+)-naloxone beyond a single dose may be required to achieve greater *Ptgs2* suppression.

Previous studies support the notion of targeting TLR4 to prevent infection-associated preterm birth. Blockade of TLR4 signaling with anti-TLR4 monoclonal antibody reduces leukocyte activation and the incidence of preterm labor induced by LPS^24^. In mice given *Fusobacterium nucleatum*, a gram negative bacteria associated with preterm birth and premature rupture of membranes in women^51^, administration of a TLR4 antagonist lipid A mimetic CXR-526 acted to reduce fetal loss^30^. CXR-526 did not suppress bacterial colonization of the placenta, but did reduce the extent of necrosis within placental tissue^30^. The authors concluded that decreased placental necrosis might reflect TLR4 antagonist-mediated suppression of pro-inflammatory cytokine expression, as reported previously for CXR-526 in a mouse model of inflammatory bowel disease^52^, but cytokines were not directly investigated in that study.

Other studies identify downstream agents of TLR4-driven inflammation as additional effective targets for inhibiting infection-induced preterm birth^53^. Experiments in null mutant mice implicate IL1 and IL6, cytokines induced by TLR4 activation of NFκB, in amplifying and accelerating the birth cascade^41^,^47^, while anti-inflammatory IL10 suppresses IL1 and IL6 production and protects against LPS-induced preterm birth^48^,^54^. As well as cytokines, immune regulators that attenuate TLR-mediated inflammation can impact susceptibility to LPS-induced preterm birth. For example, the cannabinoid receptor Cnr2 modulates dendritic cell production of IL10 and IL6, such that mice genetically deficient in Cnr2 are resistant to LPS-induced preterm birth^55^. Prostaglandins synthesized after *Ptgs2* induction by LPS administration contribute to cervical ripening and other endpoints of parturition, and suppression of *Ptgs2* with specific inhibitors can modulate LPS-induced PTD rate^56^. Pretreatment with rosiglitazone, which promotes placental synthesis of peroxisome proliferator-activated receptor-γ (PPARγ) similarly attenuates responsiveness to LPS-induced PTD, in association with suppressed NFκB signaling and reduced synthesis of TNF, IL1, IL6 and other pro-inflammatory chemokines^57^. Thus the current study adds to considerable evidence that TLR-driven inflammatory signaling can be attenuated to effectively reduce susceptibility to preterm birth.

TLR4 is an attractive drug target in preterm birth because of its role at the apex of the inflammatory pathway. (+)-Naloxone and related drug compounds have potential benefits over neutralising antibodies and other TLR4 antagonists. (+)-Naloxone potently blocks LPS-induced TLR4-mediated signaling in a variety of non-pregnancy models, suppressing NFκB activation and inhibiting TNF and IL1B induction in immune cells^34^,^35^. As well as capacity to access and cross the placental barrier^37^, the established safety profile of closely related (−)-naloxone in pregnancy and infants is encouraging. In humans, (−)-naloxone is approved for use in pregnancy and no negative neonatal outcomes are reported^58^,^59^. Given the lack of opioid receptor activity of (+)-naloxone owing to the stereo-selectivity of opioid receptors^33^, (+)-naloxone has distinct advantages over the currently available (−)-naloxone by virtue of its inability to alter endogenous opioid signaling, which in a clinical setting would afford freedom to employ exogenous opioids for maternal pain relief in labor.

Together, these results indicate that (+)-naloxone is a useful experimental drug for investigating the utility of small molecule TLR inhibitors as pharmacological agents that might be administered in association with antibiotics to contain infection and suppress progression to preterm birth^53^. While clearly there are major challenges to consider in extrapolating this work to clinical application, we speculate that such TLR4 inhibitors may have particular value as prophylactic agents in threatened preterm labour where increased TLR4 expression disposes to elevated susceptibility, even in the absence of infection^60^,^61^, or when *TLR4* SNPs associated with an increased risk of PTD and premature rupture of membranes are identified^62^,^63^. A likely advantage over current tocolytic agents is that TLR4 inhibitors act upstream of the pro-inflammatory cascade, overcoming the limitation of agents such as prostaglandin inhibitors which suppress uterine contractility, the final phase of labour, without impacting upstream pro-inflammatory activity^45^,^64^.

A key consideration is the impact on the neonate of *in utero* exposure to TLR4 inhibitors, and their effect on fetal capacity to withstand intrauterine inflammation, which is present to some extent in the majority of human preterm deliveries^8^. Sequalae of *in utero* inflammatory insult include fetal and newborn brain white matter disease, cerebral palsy, necrotizing enterocolitis, and chronic lung disease^8^, causing neurodevelopmental disability and a range of recurrent health problems in childhood^5^. Inflammation can provoke fetal brain injury even when inflammation is insufficient to activate parturition^49^, indicating the risk of sustained exposure to inflammatory mediators *in utero*. Clearly, clinical progression of this work requires extensive investigation of the benefits and risks of pharmacological delay of preterm birth for offspring, particularly effects on neurodevelopment, to ensure any treatment interventions adequately protect the fetus from inflammatory injury *in utero*.

## Materials and Methods

### Mice

C57Bl/6 (B6) mice were obtained from Laboratory Animal Services, University of Adelaide and maintained in the specific pathogen-free University of Adelaide Medical School Animal House with a 12:12 h light-dark cycle. Food and water were provided *ad libitum*. Animals were utilized in accordance with the NHMRC Australian Code of Practice for the care and use of animals for scientific purposes. All experimental protocols were approved by the University of Adelaide Animal Ethics Committee, using methods in accordance with the relevant guidelines and regulations.

One to three virgin female mice of 8–12 weeks of age were housed with a proven fertile B6 male and checked daily between 0800–1000 h for vaginal plugs, as evidence of mating. The morning of vaginal plug detection was designated gestational day (gd) 0.5. Females were removed from the male and housed individually.

### Treatments and pregnancy outcomes, i.p. LPS model

Pregnant mice were administered lipopolysaccharide (LPS, 0.5 μg; *S. typhimurium*; Sigma-Aldrich, St. Louis, MO, USA) in 200 μl PBS i.p., or PBS control, at 1100 h on gd 16.5; a protocol shown previously to induce PTD in ~50% of B6 mice^40^ ^41^. Mice were immediately administered (+)-naloxone (60 mg/kg) in 100 μl PBS + 0.1% BSA i.p., or vehicle control, within 5 min of LPS injection on gd 16.5, plus a further 3 equivalent doses at 12 h intervals on gd 17.0, 17.5 and 18.0.

Mice were monitored for evidence of delivery at 12 h intervals after the gd 16.5 treatment until gd 18.5. Vaginal bleeding or the presence of intact or partial fetal tissue, viable or non-viable pups in the cage was noted. Delivery of live or dead pups before gd 18.0 was designated PTD. At gd 18.5 all mice were killed by cervical dislocation and the intact uterus was removed and analysed as described^40^. At autopsy, maternal outcomes were classified as pregnant with viable fetuses (at least one normal fetus of >800 mg); delivered early (>1 live pup born gd 18.0–18.5), delivered preterm with live pups (>1 live pup born gd 16.5–18.0), or delivered preterm with dead pups (gd 16.5–18.0, all pups dead). The % maternal outcome in each category for each treatment group was calculated as [number]/[total pregnant mice] × 100. Fetal outcomes were classified as viable in utero (normal fetus > 800 mg), non-viable in utero (fetus hemorrhagic, anemic, malformed, or severely growth retarded < 600 mg), delivered preterm, viable (fresh implantation scar, viable pup), delivered preterm, dead (fresh implantation scar, no viable pup) or resorbed (hemorrhagic mass < 5 mm in diameter). The % fetal outcome in each category for each treatment group was calculated as [number]/[total implantation site] × 100. When viable fetuses were present, each fetus was dissected from the amniotic sac and umbilical cord, fetuses and placentae were weighed, and the fetal:placental weight ratio calculated.

### Treatment and pregnancy outcomes, in utero *E. coli* model

*E. coli* (serotype o55:K59(B5):H-) from American Type Culture Collection No. 12014 was grown overnight in 1000 ml Luria broth (LB) with shaking at 37 °C. Bacteria were washed by centrifugation in sterile PBS and killed at 100 °C for 20 min, verified by lack of growth overnight on LB agar plates. Prior to heat treatment, aliquots of live bacteria were plated on LB agar plates at 1 in 10 serial dilutions to quantify colony forming units (cfu). The heat-killed *E. coli* suspension was stored in aliquots of 1 × 10^11^ cfu/ml at −80 °C until use.

Pregnant mice were anesthetised with avertin at 1100 h on gd 16.5 and a 1.5 cm midline incision was made on the lower abdomen. Heat-killed *E. coli* (5 × 10^10^ cfu in 100 μl) or PBS was injected into the midsection of the left uterine horn at a site between two adjacent fetuses, in a treatment shown to elicit at least 50% PTD, as described^28^. The abdominal incision was closed in two layers, using sutures through the peritoneal wall and the skin. Mice were administered (+)-naloxone or vehicle as above immediately after wound closure, plus a further 3 doses at 12 h intervals as above, and allowed to recover. Females were monitored until birth for the time of parturition and the number of viable pups born, as above. Pups were weighed at 12–24 h post-delivery, at 8 days of age and at weaning.

### Cytokine and uterine activation gene expression

A second cohort of pregnant females was administered *E. coli* or vehicle, as above. The site of injection was marked by a single interrupted suture through the uterus layer prior to injection. Mice were killed by cervical dislocation 4 h post-surgery and the intact uterus of each female was removed. The two implantation sites adjacent to the injection site were each dissected into uterine myometrium (between implantation sites), entire uterine decidua (at placental attachment site), placenta and fetal membranes, and snap-frozen in liquid N_2_, then stored at −80 °C.

Tissues were homogenised using ceramic beads (Mo Bio) in Trizol (Ambion RNA, Carlsbad CA) and RNA was precipitated using isopropanol and ethanol, then DNAse-treated using Ambion DNA-*free™* Kit according to the manufacturer’s instructions. RNA purity and concentration were determined by A_260_ and A_280_ (NanoDrop, Wilmington, DE) and RNA integrity was verified by denaturing agarose electrophoresis. First strand cDNA was reverse-transcribed from 2 μg extracted RNA with Superscript III (Invitrogen Life Technologies, Carlsbad, CA) according to the manufacturer’s instructions, with 200 ng random sequence oligohexamers (Geneworks, Adelaide, Australia) and 500 ng oligo dT_18_ (Proligo, Lismore, Australia) at 52 °C for 1 h. Each reaction contained 2 μL of cDNA (10 ng/μL) and 18 μL of master mix consisting of Power SYBR^®^ Green PCR Master Mix (Life Technologies, Carlsbad, CA), 0.5–1 μM of 5′ and 3′ primers (listed in Supplementary Table S1) and RNase free water. PCR reactions were 10 min at 95 °C followed by 40 cycles of 95 °C for 15 sec and 60 °C for 45 sec, using a Rotorgene 6000 (Corbett Life Sciences, Sydney, Australia). PCR product integrity was confirmed by High Resolution Melt analysis. Data were normalized to β-actin mRNA expression and expressed as ΔΔCT using the formula mRNA level = Log_2_ - (Ct_*Bactin*_ − Ct_*target gene*_).

### Statistical analysis

All statistical analysis was conducted using SPSS for Windows, version 20.0 software (SPSS Inc, Chicago, IL). Data were tested for normality using a Shapiro–Wilk test. ANOVA and post hoc T-tests were used when data were normally distributed. Kruskal-Wallis and Mann-Whitney U-test were used when data were not normally distributed. Pregnancy outcome and fetal outcome data expressed as percentage were compared by χ^2^ analysis. Gestation length and viable litter size data expressed as mean ± standard error of the mean (SEM) are analyzed by two-way ANOVA and post-hoc LSD test. Fetal weight, placental weight, fetal:placental weight ratio, growth trajectory and body composition data is expressed as estimated marginal means ± SEM and analysed by Mixed Model Linear Repeated Measures ANOVA and post-hoc LSD t-test, with mother as subject. Growth curve data was also analysed by Area Under the Curve (AUC) analysis in R studio. qPCR data are mean ± SEM analyzed by one-way ANOVA and post-hoc Sidak t-test (to adjust for multiple comparisons). Differences between groups were considered significant when of *p* < 0.05.

## Additional Information

**How to cite this article**: Chin, P. Y. *et al.* Novel Toll-like receptor-4 antagonist (+)-naloxone protects mice from inflammation-induced preterm birth. *Sci. Rep.* **6**, 36112; doi: 10.1038/srep36112 (2016).

**Publisher’s note**: Springer Nature remains neutral with regard to jurisdictional claims in published maps and institutional affiliations.

## Supplementary Material

Supplementary Information
