# Supplementary material for: Novel Toll-like receptor-4 antagonist (+)-naloxone protects mice from inflammation-induced preterm birth
Source: Sci Rep. 2016 Nov 7;6:36112. doi: 10.1038/srep36112 (PMC5098167; doi:10.1038/srep36112)
Supplement: Supplementary Information [file srep36112-s1.doc]

**SUPPLEMENTARY INFORMATION**

**Novel Toll-like receptor-4 antagonist (+)-naloxone protects mice from inflammation-induced preterm birth**

Peck Yin Chin^1^, Camilla Dorian^1^, Mark R. Hutchinson^2,5^, David M. Olson^4^, Kenner C. Rice^3^, Lachlan M. Moldenhauer^1^ and Sarah A. Robertson^1^

^1^Robinson Research Institute and Adelaide Medical School, University of Adelaide, Adelaide, SA 5005, Australia.

^2^Adelaide Medical School, University of Adelaide, Adelaide, SA 5005, Australia.

^3^Chemical Biology Research Branch, National Institute on Drug Abuse and National Institute on Alcohol Abuse and Alcoholism, National Institutes of Health, Rockville, MD 20892, USA

^4^Departments of Obstetrics & Gynecology, Pediatrics and Physiology, University of Alberta, Edmonton, Alberta T6G2S2, Canada.

^5^Australian Research Council Centre of Excellence for Nanoscale BioPhotonics, Adelaide, SA, 5005, Australia

**SUPPLEMENTARY TABLE S1.** Primers for qPCR analysis of mRNA expression in reproductive and gestational tissues.

| Gene | Forward and Reverse Prime Sequence | GeneBank accession # |
| --- | --- | --- |
| *Il1a* | F-*5’* *CCGACCTCATTTTCTTCTGG* *3’,* R-*5’ GTGCACCCGACTTTGTTCTT* *3’* | NM_010554.4 |
| *Il1b* | F-*5’* CCAAAGCAATACCCAAAGAAA *3’* R-*5’* GCTTGTGCTCTGCTTGTGAG *3’* | NM 008361.3 |
| *Il6* | F-*5’ ACAACCACGGCCTTCCCTAC 3’,* R-*5’ TCCACGATTTCCCAGAGAACA 3’* | NM 031168.1 |
| *Il10*  (CSIF) | F-*5’ AGGCGCTGTCATCGATTTCT 3’* R-*5’ TGGCCTTGTAGACACCTTGGT 3’* | NM 010548.2 |
| *Tnf*  (TNFα) | F-*5’ GTAGCCCACGTCGTA 3’* R-*5’ TCCACGATTTCCCAG 3’* | NM 013693.3 |
| *Ptgs2* (Cox2) | F-*5’*  *GTTTGCATTCTTTGCCCAGC 3’* R-*5’* *AGTCCACTCCATGGCCCAGT 3’* | NM 011198.3 |
| *Actb* (β-actin) | F-*5’ CGTGGGCCGCCCTAGGCACCA 3’* R-*5’* *ACACGCAGCTCATTGTA 3’* | [NM 007393.3](http://www.ncbi.nlm.nih.gov/nucleotide/145966868?report=genbank&log$=nucltop&blast_rank=4&RID=K1X52CXF014) |
